# Case Report: Hyperthyroidism induced liver failure with Wolff–Parkinson–White syndrome

**DOI:** 10.3389/fmed.2026.1794028

**Published:** 2026-03-27

**Authors:** Yifan Ke, Yuankai Wu, Yutian Chong

**Affiliations:** Department of Infectious Diseases, The Third Affiliated Hospital of Sun Yat-sen University, Guangzhou, China

**Keywords:** antithyroid drugs, case report, hyperthyroidism, liver failure, radiofrequency ablation, Wolff–Parkinson–White syndrome

## Abstract

Liver failure is a critical condition secondary to not only hepatopathy but also extrahepatic etiologies. This article presents a unique case of hyperthyroidism-induced liver failure complicated with Wolff–Parkinson–White syndrome and pneumonia. The 53-year-old male patient had a history of hepatitis B but no prior hyperthyroidism or medication use. He was admitted with jaundice, fever, and palpitations, and underwent one session of therapeutic plasma exchange in the intensive care unit. Other treatments included hepatoprotective agents, blood component transfusions, antithyroid drugs, antiarrhythmic therapy, and antibiotics. Radiofrequency catheter ablation was performed to eliminate an accessory atrioventricular pathway. Within 3 weeks, significant improvement was observed in hepatic, coagulation, thyroid, and cardiac functions. After 6 weeks of intensive care, the patient was transferred to a general ward and discharged on day 52. Follow-up confirmed full recovery without sequelae. In conclusion, early antithyroid drugs and radiofrequency catheter ablation are safe and effective in this case, while radioiodine therapy and glucocorticoids are not essential. Accurate diagnosis and etiological treatment are the keys to managing complex liver failure cases in clinical practice.

## 1 Introduction

The main causes of liver failure (LF) are viral hepatitis in the Asia-Pacific region and hepatotoxic substances such as drugs and alcohol in Western countries, respectively (1). Besides, patients with LF of unknown etiology are frequently encountered in clinical practice. Hyperthyroidism-induced liver injury (HILI) commonly occurs in patients with uncontrolled thyroid function or pre-existing liver disease. Its incidence and pathogenesis remain unclear but may be associated with an increased metabolic rate and oxygen consumption. This can cause relative hypoxia in the perivenular region, subsequently triggering hepatocyte apoptosis and oxidative stress (2). Although HILI is common in clinical practice, its progression to LF is rarely reported. The relevant diagnostic and therapeutic criteria have not yet been established.

This article reports a case of hyperthyroidism-induced LF following HBsAg seroclearance. The patient also had Wolff–Parkinson–White (WPW) syndrome, which is characterized by an accessory atrioventricular conduction pathway resulting from congenital heart anomalies. Its episodes can induce tachyarrhythmia and, in severe cases, may cause palpitations, chest tightness, or even heart failure (3). We believe this case provides valuable insights into the etiological diagnosis and treatment of LF and serves as a practical reference for physicians managing similar patients.

## 2 Case description

The 53-year-old male patient had no history of medication use or alcohol abuse. He had been diagnosed with acute hepatitis B 30 years prior and achieved spontaneous HBsAg seroconversion 8 years ago. He also had an 18-year history of WPW syndrome. He denied other family history, but his father also suffered from hepatitis B. He developed persistent dull pain in the upper abdomen 2 weeks prior accompanied by frequent diarrhea. Liver function tests revealed elevated alanine aminotransferase [ALT, 6,153 U/L, reference range (RR): 5–40], aspartate aminotransferase (AST, 8,255 U/L, RR: 9–48), and total bilirubin (TB, 110.2 μmol/L, RR: 2–20). Coagulation tests revealed elevated prothrombin time (PT, 23.3 s, RR: 11–14.6) and international normalized ratio (INR, 4.25). The white blood cell count (WBC) was 12.8 × 10^9^/L (RR: 4–10). He received anti-infective (cefoperazone-sulbactam), hepatoprotective (reduced glutathione, magnesium isoglycyrrhizinate, and compound amino acid injection), and symptomatic therapy at a local hospital. During this period, he experienced one episode of supraventricular tachycardia, which was controlled with amiodarone and electrical cardioversion. Subsequently, he underwent one session of therapeutic plasma exchange (TPE) in the ICU. Four days prior, he developed recurrent fever (maximum temperature 38.5 °C) accompanied by delirium and palpitations. A chest CT scan showed bilateral pneumonia and right-sided pleural effusion. Although the ALT, AST, and WBC had normalized, the TB continued to rise to 219.9 μmol/L. Therefore, he was transferred to our center for further management.

**Figure 1:**
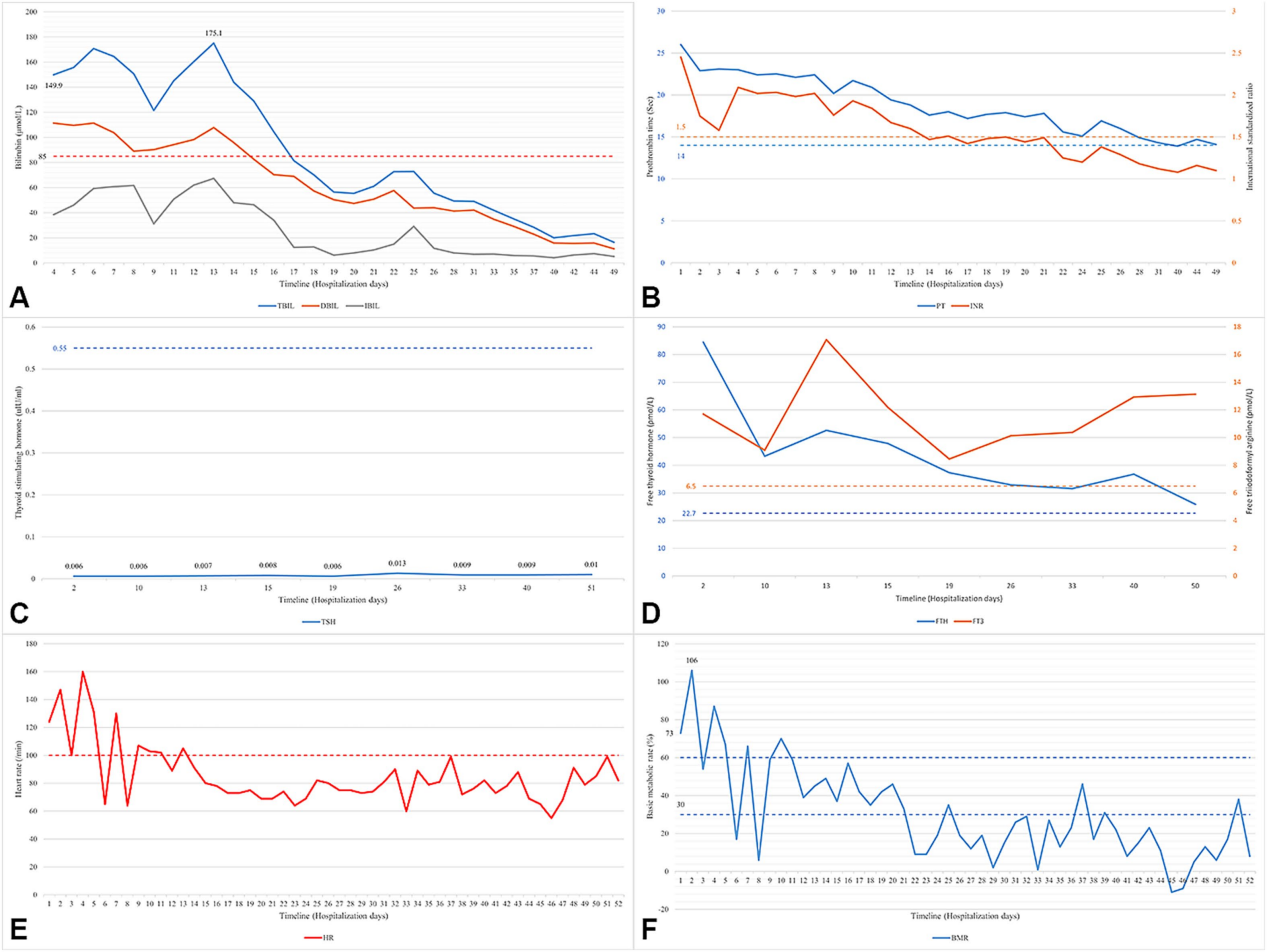
Case description. **(A)** Serum bilirubin; **(B)** coagulation function; **(C)** thyroid stimulating hormone; **(D)** free thyroxine; **(E)** heart rate; **(F)** basic metabolic rate. TBIL, total bilirubin; DBIL, direct bilirubin; IBIL, indirect bilirubin; PT, prothrombin time; INR, international standardized ratio; TSH, thyroid stimulating hormone; FT4, free thyroxine; FT3, free triiodothyronine; HR, heart rate; BMR, basic metabolic rate.

On admission, the patient was conscious with a temperature of 37.6 °C, a heart rate (HR) of 175 beats per minute, and a blood pressure of 140/80 mmHg. Physical examination revealed jaundice, palmar erythema, spider angiomas, and multiple subcutaneous ecchymoses. Bilateral breath sounds were diminished. The thyroid gland was enlarged with an associated vascular bruit. Trends in key laboratory findings and vital signs are shown in Figure 1. The baseline thyroid function tests showed a suppressed thyroid-stimulating hormone level of 0.006 μIU/mL (RR: 0.55–4.78), with elevated free thyroxine (84.52 ρmol/L, RR: 11.5–22.7) and free triiodothyronine (11.7 ρmol/L, RR: 3.5–6.5) levels. Thyroid peroxidase antibody and thyrotropin receptor antibody levels were 529.6 U/mL (RR: 0–60) and 14.21 U/L (RR: <12), respectively. The B-type natriuretic peptide (BNP) level was 2,550 ρg/mL (RR: <100). Electrocardiograms showed supraventricular tachycardia and atrial fibrillation with a HR exceeding 250 beats per minute. Abdominal ultrasonography showed a coarse and heterogeneous liver parenchymal echotexture with peritoneal effusion. Cardiac ultrasonography showed atrial enlargement, mitral and tricuspid regurgitation, and a left ventricular ejection fraction (LVEF) of 59%. The initial diagnoses were LF, Graves’ disease, hyperthyroid heart disease (NYHA class III), WPW syndrome, and pneumonia.

For hyperbilirubinemia, intravenous polyene phosphatidylcholine, adenosylmethionine butane disulfonate, and reduced glutathione were administered. The patient’s TB level reduced below 100 μmol/L by hospital day 17 and normalized by day 40. For coagulopathy, fresh frozen plasma and cryoprecipitate were transfused repeatedly during the first 2 weeks. The PT shortened significantly, and the INR normalized by hospital day 16. For hyperthyroidism, oral methimazole was started at 20 mg daily and later increased to 30 mg daily. After 3 weeks, the basic metabolic rate had largely normalized, although FT4 and FT3 levels remained on the high side. For arrhythmias, intravenous propafenone and oral propranolol were administered during the first week, but palpitations recurred frequently. On hospital day 9, we performed an intracardiac electrophysiological study and radiofrequency catheter ablation. An accessory pathway at the right tricuspid annulus was completely ablated. On the post-procedure day 2, the rhythm converted to sinus and the HR normalized. For cardiac insufficiency, continuous intravenous lyophilized recombinant human brain natriuretic peptide was administered via micro-infusion pump, supplemented with oral digoxin and spironolactone. After 3 weeks, the BNP level decreased to 442 ρg/mL, and the natriuretic peptide infusion was discontinued. For pneumonia, intravenous tigecycline and cefoperazone-sulbactam were empirically administered. On hospital day 9, *Burkholderia cepacia* was isolated from the sputum culture. Based on antimicrobial susceptibility testing, the regimen was changed to intravenous meropenem. By hospital day 20, a chest X-ray showed complete resolution of bilateral pneumonia, and antibiotics were discontinued.

**Figure 2:**
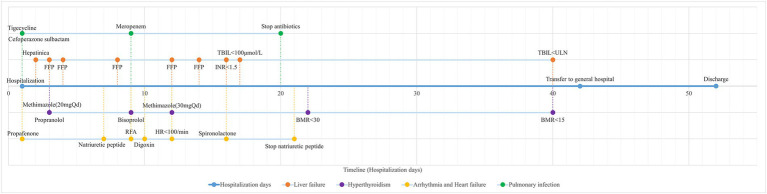
Timeline of hospitalization. TBIL, total bilirubin; INR, international standardized ratio; HR, heart rate; BMR, basic metabolic rate; FFP, fresh frozen plasma; RFA, radiofrequency ablation.

After 3 weeks of intensive care, the patient’s hepatic, coagulation, thyroid, and cardiac functions showed marked improvement, and arrhythmia and pneumonia were simultaneously controlled. Following maintenance therapy and symptomatic supportive care, he was transferred to a general ward on hospital day 42 and discharged on day 52. The treatment and recovery timeline is summarized in Figure 2. One month after discharge, follow-up examinations including complete blood count, liver and kidney function tests, electrolytes, and coagulation profile revealed no significant abnormalities.

## 3 Discussion

Identifying the primary etiology is fundamental to treating LF. A previous study identified age >45 years, HR >90 beats per minute, and free thyroid hormone >3 times the upper limit of normal as independent risk factors for HILI, which were consistent with the characteristics of this case (4). However, Graves’ disease was undiagnosed before the patient was transferred to our center. Although the patient initially presented with liver injury, arrhythmia, and fever, these manifestations could be attributed to his history of hepatitis B, WPW syndrome, and pneumonia, respectively. Therefore, the etiological investigation of LF should be more comprehensive.

Potential etiologies of HILI include: direct hepatotoxicity from excessive thyroid hormones, hepatocellular degeneration due to accelerated glycogen and protein catabolism, congestive hepatocyte necrosis, pre-existing liver disease, drug-induced liver injury, and autoimmune liver injury (5). Among these, anti-thyroid drugs (ATDs) can induce acute LF. Jaundice and coagulopathy manifested in this patient prior to ATD initiation, thereby excluding this etiology. We also tested a panel of autoantibodies, including anti-nuclear (ANA), anti-mitochondrial (AMA), anti-neutrophil cytoplasmic (ANCA), anti-liver kidney microsomal type 1 (anti-LKM1), anti-liver cytosol type 1 (anti-LC1), and anti-glycoprotein 210 (anti-GP210) antibodies, to rule out autoimmune diseases. In addition to the direct hepatotoxicity of thyroid hormones, cardiac insufficiency likely contributed to LF in this patient. Although WPW syndrome does not induce liver injury, the extremely high HR which exceeded that for hyperthyroid heart disease, likely exacerbated cardiac insufficiency during episodes (3). Furthermore, despite being HBsAg negative, the patient had physical and ultrasound signs of hepatopathy, suggesting that post-hepatitis B liver injury was a potential cause.

In LF treatment, hepatoprotective agents and blood component transfusion have definite curative effect (6). However, eliminating the primary etiology is more crucial. The patient underwent one session of TPE. Although primarily aimed at reducing TB levels, TPE also removed excessive thyroid hormones (7). The patient met the criteria for thyroid storm based on both the Burch-Wartofsky Point Scale (>45) and the Japan Thyroid Association criteria (TS1) (8). However, glucocorticoids were not administered to him, nor was TPE repeated, unlike in other HILI cases (9–11). Oral methimazole was administered immediately because the transaminase levels had normalized on admission. Although ATDs have hepatotoxicity, methimazole carries a lower risk than propylthiouracil (12). Oral propranolol helps control HR and inhibit the peripheral conversion of free tetraiodothyronine to triiodothyronine (13). Some studies suggest that ^131^I therapy may be preferable to ATDs in patients with HILI (14). However, a retrospective study found no significant survival difference between methimazole monotherapy and artificial liver support combined with ^131^I therapy in patients with hyperthyroidism and LF (7).

Hyperthyroid heart disease typically presents as atrial fibrillation. However, concurrent WPW syndrome resulted in both supraventricular tachycardia and atrial fibrillation (15). Despite the presence of LF, we proceeded with radiofrequency catheter ablation because the pharmacological treatment had failed to control the arrhythmia. This decisive intervention accelerated recovery in the early stage.

In summary, we successfully managed a complex case of hyperthyroidism-induced LF complicated with WPW syndrome and pneumonia. The patient achieved complete recovery without sequelae. The key insight was that a targeted therapeutic strategy based on accurate diagnosis is crucial. Glucocorticoids and ^131^I therapy are commonly used but not essential in HILI. ATDs and radiofrequency catheter ablation proved safe and effective in this case.

## 4 The patient’s perspective

I had always been in good health, yet the sudden onset of abdominal pain, jaundice, palpitations, and fever nearly cost me my life. I was hospitalized for more than 2 months in total, first at a local hospital and then at the Third Affiliated Hospital of Sun Yat-sen University, during which I also received over 6 weeks of intensive care treatment. Following a series of examinations and evaluations, my attending physician finally confirmed that hyperthyroidism was the primary cause of my liver failure. Subsequently, I underwent comprehensive treatment involving a variety of oral medications, intravenous preparations, and surgical intervention. Fortunately, under the careful planning and close monitoring of the medical staff, I did not experience any severe adverse reactions. I eventually recovered smoothly and am now able to live a normal life without any sequelae. I consider that the diagnosis and treatment provided by Dr. Chong’s team were accurate and effective. They successfully saved my life at the minimum cost, for which I am deeply grateful.

## Data Availability

The original contributions presented in the study are included in the article/supplementary material, further inquiries can be directed to the corresponding author.
